# 1-(4-Amino-3,5-dichloro­phen­yl)ethanol

**DOI:** 10.1107/S1600536811024196

**Published:** 2011-06-30

**Authors:** Peng Liu, Jian-Feng Wu, Ping-An Wang, Wei He, Hai-Bo Wang

**Affiliations:** a Department of Chemistry, School of Pharmacy, Fourth Military Medical University, Changle West Road 169, 710032 Xi-An, People’s Republic of China

## Abstract

The asymmetric unit of the title compound, C_8_H_9_Cl_2_NO, contains two crystallographically independent mol­ecules which are connected *via* an N—H⋯O hydrogen bond . There is aromatic π–π stacking in the crystal, with a centroid–centroid distance between benzene rings of 3.48 (2)Å. The crystal packing is stabilized by intermolecular hydrogen bonds.

## Related literature

For the synthetic use of the title compound and related compounds, see: Judkins *et al.* (1991 ▶); Ehrhardt (1990 ▶); Kelser (2007 ▶); Lu (2001 ▶); Pri-Bar *et al.* (1990 ▶); Shukrallah *et al.* (2004 ▶).
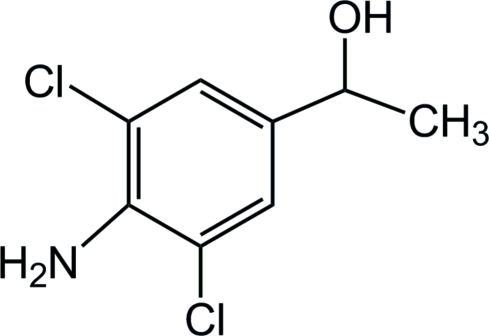


## Experimental

### 

#### Crystal data

C_8_H_9_Cl_2_NO
*M* *_r_* = 206.06
Monoclinic, 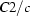

*a* = 16.472 (2) Å
*b* = 16.110 (2) Å
*c* = 14.6756 (19) Å
β = 107.049 (2)°
*V* = 3723.2 (8) Å^3^
*Z* = 16
Mo *K*α radiation
μ = 0.65 mm^−1^
*T* = 296 K
0.15 × 0.13 × 0.08 mm

#### Data collection

Bruker APEXII CCD diffractometer
Absorption correction: multi-scan (*SADABS*; Bruker, 2007 ▶) *T*_min_ = 0.909, *T*_max_ = 0.950
9273 measured reflections
3279 independent reflections
2855 reflections with *I* > 2σ(*I*)
*R*_int_ = 0.017

#### Refinement

*R*[*F* ^2^ > 2σ(*F* ^2^)] = 0.032
*wR*(*F* ^2^) = 0.128
*S* = 1.06
3279 reflections
277 parameters
1 restraint
H atoms treated by a mixture of independent and constrained refinement
Δρ_max_ = 0.42 e Å^−3^
Δρ_min_ = −0.46 e Å^−3^

### 

Data collection: *APEX2* (Bruker, 2007 ▶); cell refinement: *SAINT* (Bruker, 2007 ▶); data reduction: *SAINT*; program(s) used to solve structure: *SHELXS97* (Sheldrick, 2008 ▶); program(s) used to refine structure: *SHELXL97* (Sheldrick, 2008 ▶); molecular graphics: *ORTEP-3* (Farrugia, 1997 ▶) and *Mercury* (Macrae *et al.*, 2006 ▶); software used to prepare material for publication: *publCIF* (Westrip, 2010) ▶.

## Supplementary Material

Crystal structure: contains datablock(s) I, global. DOI: 10.1107/S1600536811024196/zk2012sup1.cif

Structure factors: contains datablock(s) I. DOI: 10.1107/S1600536811024196/zk2012Isup2.hkl

Supplementary material file. DOI: 10.1107/S1600536811024196/zk2012Isup3.cml

Additional supplementary materials:  crystallographic information; 3D view; checkCIF report

## Figures and Tables

**Table 1:** Hydrogen-bond geometry (Å, °)

*D*—H⋯*A*	*D*—H	H⋯*A*	*D*⋯*A*	*D*—H⋯*A*
N1—H13⋯O2^i^	0.82 (3)	2.17 (3)	2.927 (3)	154 (3)
O1—H16⋯N1^ii^	0.83 (3)	2.12 (3)	2.941 (3)	169 (3)
O2—H18⋯N2^iii^	0.79 (3)	2.13 (3)	2.912 (3)	172 (2)

Symmetry codes: (i) 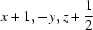; (ii) 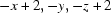; (iii) 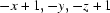.

**Fig. 1.**
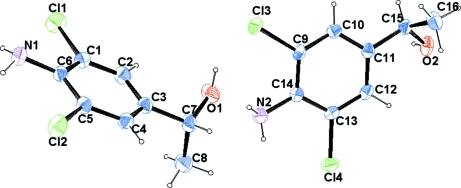
The molecular structure of the title compound, with atom labels and anisotropic displacement ellipsoids drawn at 50% probability level.

## supplementary crystallographic information

### Comment

We report here the crystal structure of the title compound 1-(4-amino-3, 5-dichlorophenyl) ethanol (I), a typical secondary aromatic alcohol and an important organic building block (Fig. 1). Bond lengths and angles are within normal ranges. As part of our ongoing studies of the secondary aromatic alcohol, the title compound was synthesized and characterized by X-ray diffraction. The asymmetric unit of the title compound (I) contains two crystallographically independent molecules in which both molecules are connected *via* N—H···O hydrogen bonds. The dihedral angle between the two benzene rings is 60.49°. The packing of molecules in the crystal structure is stabilized by intermolecular O—H···N hydrogen bonds.

### Experimental

1-(4-amino-3,5-dichlorophenyl)ethanone (1.03 g,5 mmol) was dissolved in anhydrous methanol(25 ml) at room temperature, followed by addition of NaBH4(0.227 g, 6 mmol). The mixture was stirred vigorously at room temperature until TLC showed no ethanone. The solvent was evaporated to dryness under reduced pressure to obtain a crude product, which was purified by a flash column chromatography (n-hexane/EtOAc 8:1) to afford pure colorless compound in 89.6% yield. Then, the title compound (40 mg,0.19 mmol) was dissolved in ethyl acetate/n-hexane (7:3,15 ml). Colorless crystals were isolated after several days.

### Refinement

In both structures all the H atoms were discernible in the difference Fourier maps. However, they were constrained by riding model approximation. *C*—H_methyl_=0.96 Å; *C*—H_aryl_=0.93 Å; *U*_iso_H_methyl_ and *U*_iso_H_aryl_ are 1.5 U _eq_(C) and 1.2 U _eq_ (C), respectively.

### Crystal data

**Table:**

C_8_H_9_Cl_2_NO	*F*(000) = 1696
*M**_r_* = 206.06	*D*_x_ = 1.470 Mg m^−^^3^
Monoclinic, *C*2/*c*	Mo *K*α radiation, λ = 0.71073 Å
*a* = 16.472 (2) Å	Cell parameters from 5355 reflections
*b* = 16.110 (2) Å	θ = 2.6–28.3°
*c* = 14.6756 (19) Å	µ = 0.65 mm^−^^1^
β = 107.049 (2)°	*T* = 296 K
*V* = 3723.2 (8) Å^3^	Block, colorless
*Z* = 16	0.15 × 0.13 × 0.08 mm

### Data collection

**Table:**

Bruker APEXII CCD diffractometer	3279 independent reflections
Radiation source: fine-focus sealed tube	2855 reflections with *I* > 2σ(*I*)
graphite	*R*_int_ = 0.017
φ and ω scans	θ_max_ = 25.0°, θ_min_ = 1.8°
Absorption correction: multi-scan (*SADABS*; Bruker, 2007)	*h* = −19→15
*T*_min_ = 0.909, *T*_max_ = 0.950	*k* = −19→17
9273 measured reflections	*l* = −17→16

### Refinement

**Table:**

Refinement on *F*^2^	Primary atom site location: structure-invariant direct methods
Least-squares matrix: full	Secondary atom site location: difference Fourier map
*R*[*F*^2^ > 2σ(*F*^2^)] = 0.032	Hydrogen site location: inferred from neighbouring sites
*wR*(*F*^2^) = 0.128	H atoms treated by a mixture of independent and constrained refinement
*S* = 1.06	*w* = 1/[σ^2^(*F*_o_^2^) + (0.1*P*)^2^] where *P* = (*F*_o_^2^ + 2*F*_c_^2^)/3
3279 reflections	(Δ/σ)_max_ = 0.001
277 parameters	Δρ_max_ = 0.42 e Å^−^^3^
1 restraint	Δρ_min_ = −0.46 e Å^−^^3^

### Special details

**Table:**

Geometry. All e.s.d.'s (except the e.s.d. in the dihedral angle between two l.s. planes) are estimated using the full covariance matrix. The cell e.s.d.'s are taken into account individually in the estimation of e.s.d.'s in distances, angles and torsion angles; correlations between e.s.d.'s in cell parameters are only used when they are defined by crystal symmetry. An approximate (isotropic) treatment of cell e.s.d.'s is used for estimating e.s.d.'s involving l.s. planes.
Refinement. Refinement of *F*^2^ against ALL reflections. The weighted *R*-factor *wR* and goodness of fit *S* are based on *F*^2^, conventional *R*-factors *R* are based on *F*, with *F* set to zero for negative *F*^2^. The threshold expression of *F*^2^ > σ(*F*^2^) is used only for calculating *R*-factors(gt) *etc*. and is not relevant to the choice of reflections for refinement. *R*-factors based on *F*^2^ are statistically about twice as large as those based on *F*, and *R*- factors based on ALL data will be even larger.

### Fractional atomic coordinates and isotropic or equivalent isotropic displacement parameters (Å^2^)

**Table:**

	*x*	*y*	*z*	*U*_iso_*/*U*_eq_	
Cl1	1.10844 (4)	0.04952 (4)	0.86690 (4)	0.0620 (2)	
Cl2	1.09417 (4)	0.16958 (4)	1.20328 (4)	0.0598 (2)	
Cl3	0.70719 (3)	−0.07065 (4)	0.67509 (4)	0.0557 (2)	
Cl4	0.44673 (4)	0.14455 (3)	0.61899 (4)	0.0527 (2)	
O1	0.78216 (11)	0.06516 (11)	0.85101 (13)	0.0617 (5)	
O2	0.31701 (9)	−0.14705 (11)	0.48677 (12)	0.0519 (4)	
N1	1.18071 (11)	0.10254 (12)	1.06839 (15)	0.0468 (4)	
N2	0.63069 (12)	0.09603 (13)	0.67790 (13)	0.0440 (4)	
C1	1.04879 (13)	0.08619 (12)	0.93915 (14)	0.0416 (5)	
C2	0.96140 (14)	0.08983 (14)	0.90409 (15)	0.0458 (5)	
C3	0.91343 (12)	0.11733 (12)	0.96181 (14)	0.0414 (5)	
C4	0.95554 (13)	0.14186 (12)	1.05372 (15)	0.0408 (5)	
C5	1.04275 (12)	0.13832 (12)	1.08668 (13)	0.0389 (4)	
C6	1.09322 (12)	0.11026 (11)	1.03173 (13)	0.0375 (4)	
C7	0.81676 (14)	0.12321 (15)	0.92535 (18)	0.0517 (5)	
C8	0.78811 (16)	0.20783 (16)	0.8855 (2)	0.0712 (8)	
H8A	0.7272	0.2094	0.8632	0.107*	
H8B	0.8106	0.2193	0.8334	0.107*	
H8C	0.8082	0.2489	0.9344	0.107*	
C9	0.59864 (12)	−0.05077 (12)	0.64539 (13)	0.0377 (4)	
C10	0.54210 (12)	−0.11507 (13)	0.61455 (14)	0.0388 (4)	
C11	0.45556 (12)	−0.10121 (12)	0.58610 (13)	0.0380 (4)	
C12	0.42693 (12)	−0.02043 (13)	0.58929 (14)	0.0406 (5)	
C13	0.48443 (12)	0.04327 (12)	0.62044 (13)	0.0366 (4)	
C14	0.57235 (11)	0.03095 (12)	0.65081 (12)	0.0354 (4)	
C15	0.39451 (13)	−0.17286 (14)	0.55311 (16)	0.0458 (5)	
C16	0.37270 (17)	−0.21407 (17)	0.6356 (2)	0.0578 (6)	
H1	0.3668 (13)	−0.0092 (11)	0.5668 (14)	0.035 (5)*	
H2	0.5640 (15)	−0.1665 (14)	0.6168 (17)	0.048 (6)*	
H3	0.9238 (15)	0.1595 (14)	1.0923 (18)	0.048 (6)*	
H4	0.4188 (13)	−0.2126 (14)	0.5260 (15)	0.045 (6)*	
H5	0.9332 (14)	0.0707 (13)	0.8489 (16)	0.045 (6)*	
H6	0.3307 (18)	−0.2551 (16)	0.612 (2)	0.069 (7)*	
H8	0.6703 (16)	0.0815 (14)	0.7248 (17)	0.045 (6)*	
H9	0.3513 (16)	−0.1676 (16)	0.6741 (18)	0.058 (7)*	
H10	0.4207 (17)	−0.2342 (17)	0.6731 (19)	0.062 (7)*	
H11	0.6117 (18)	0.1357 (18)	0.6943 (19)	0.061 (9)*	
H12	0.7895 (15)	0.1125 (15)	0.9809 (17)	0.051 (6)*	
H13	1.2082 (18)	0.1076 (17)	1.030 (2)	0.062 (8)*	
H15	1.2046 (18)	0.1337 (17)	1.118 (2)	0.062 (8)*	
H16	0.794 (2)	0.016 (2)	0.867 (2)	0.084 (10)*	
H18	0.3276 (19)	−0.1363 (17)	0.4388 (16)	0.070 (9)*	

### Atomic displacement parameters (Å^2^)

**Table:**

	*U*^11^	*U*^22^	*U*^33^	*U*^12^	*U*^13^	*U*^23^
Cl1	0.0664 (4)	0.0764 (4)	0.0496 (4)	0.0058 (3)	0.0267 (3)	−0.0065 (3)
Cl2	0.0441 (3)	0.0892 (5)	0.0391 (3)	0.0033 (3)	0.0011 (2)	−0.0144 (3)
Cl3	0.0275 (3)	0.0586 (4)	0.0742 (4)	0.0044 (2)	0.0044 (2)	−0.0059 (3)
Cl4	0.0508 (4)	0.0457 (3)	0.0588 (4)	0.0116 (2)	0.0116 (3)	−0.0049 (2)
O1	0.0475 (9)	0.0491 (10)	0.0669 (11)	−0.0050 (7)	−0.0171 (8)	−0.0017 (8)
O2	0.0332 (8)	0.0783 (11)	0.0422 (9)	−0.0106 (7)	0.0082 (6)	−0.0022 (7)
N1	0.0341 (9)	0.0582 (12)	0.0472 (11)	−0.0009 (8)	0.0106 (8)	−0.0004 (9)
N2	0.0387 (10)	0.0460 (10)	0.0430 (10)	−0.0016 (8)	0.0053 (8)	−0.0039 (8)
C1	0.0448 (11)	0.0432 (11)	0.0381 (10)	0.0021 (9)	0.0142 (9)	0.0011 (8)
C2	0.0463 (12)	0.0497 (12)	0.0345 (11)	−0.0036 (9)	0.0010 (9)	−0.0027 (9)
C3	0.0345 (10)	0.0410 (10)	0.0432 (11)	−0.0028 (8)	0.0027 (8)	0.0011 (8)
C4	0.0359 (11)	0.0431 (11)	0.0418 (11)	0.0021 (8)	0.0089 (9)	−0.0019 (8)
C5	0.0369 (10)	0.0421 (11)	0.0333 (10)	−0.0026 (8)	0.0033 (8)	−0.0008 (8)
C6	0.0337 (10)	0.0377 (10)	0.0395 (10)	0.0011 (7)	0.0082 (8)	0.0054 (8)
C7	0.0368 (11)	0.0531 (12)	0.0561 (13)	−0.0047 (9)	−0.0009 (10)	0.0000 (10)
C8	0.0454 (14)	0.0526 (14)	0.101 (2)	0.0025 (11)	−0.0017 (13)	0.0039 (13)
C9	0.0269 (9)	0.0488 (11)	0.0348 (10)	0.0023 (8)	0.0050 (7)	0.0024 (8)
C10	0.0365 (10)	0.0404 (11)	0.0379 (10)	0.0041 (8)	0.0086 (8)	0.0008 (8)
C11	0.0341 (10)	0.0448 (11)	0.0345 (10)	−0.0027 (8)	0.0094 (8)	−0.0022 (8)
C12	0.0282 (10)	0.0541 (12)	0.0383 (10)	0.0026 (8)	0.0081 (8)	−0.0010 (9)
C13	0.0363 (10)	0.0413 (10)	0.0323 (9)	0.0073 (8)	0.0101 (8)	0.0007 (8)
C14	0.0331 (10)	0.0432 (10)	0.0283 (9)	−0.0013 (8)	0.0068 (7)	0.0001 (8)
C15	0.0352 (11)	0.0500 (12)	0.0525 (13)	−0.0036 (9)	0.0132 (9)	−0.0109 (10)
C16	0.0518 (15)	0.0547 (14)	0.0610 (15)	−0.0106 (12)	0.0073 (12)	0.0077 (12)

### Geometric parameters (Å, °)

**Table:**

Cl1—C1	1.7463 (19)	C4—H3	0.92 (2)
Cl2—C5	1.7462 (19)	C5—C6	1.392 (3)
Cl3—C9	1.7415 (19)	C7—C8	1.504 (3)
Cl4—C13	1.7437 (19)	C7—H12	1.05 (2)
O1—C7	1.423 (3)	C8—H8A	0.9600
O1—H16	0.84 (3)	C8—H8B	0.9600
O2—C15	1.422 (3)	C8—H8C	0.9600
O2—H18	0.792 (17)	C9—C10	1.378 (3)
N1—C6	1.388 (3)	C9—C14	1.395 (3)
N1—H13	0.82 (3)	C10—C11	1.381 (3)
N1—H15	0.87 (3)	C10—H2	0.90 (2)
N2—C14	1.399 (3)	C11—C12	1.390 (3)
N2—H8	0.83 (3)	C11—C15	1.514 (3)
N2—H11	0.78 (3)	C12—C13	1.380 (3)
C1—C2	1.381 (3)	C12—H1	0.96 (2)
C1—C6	1.396 (3)	C13—C14	1.399 (3)
C2—C3	1.389 (3)	C15—C16	1.513 (3)
C2—H5	0.86 (2)	C15—H4	0.91 (2)
C3—C4	1.381 (3)	C16—H6	0.95 (3)
C3—C7	1.527 (3)	C16—H9	1.06 (3)
C4—C5	1.376 (3)	C16—H10	0.88 (3)
			
C7—O1—H16	113 (2)	H8A—C8—H8B	109.5
C15—O2—H18	106 (2)	C7—C8—H8C	109.5
C6—N1—H13	116 (2)	H8A—C8—H8C	109.5
C6—N1—H15	115.7 (18)	H8B—C8—H8C	109.5
H13—N1—H15	108 (3)	C10—C9—C14	122.46 (17)
C14—N2—H8	109.3 (16)	C10—C9—Cl3	119.32 (15)
C14—N2—H11	113 (2)	C14—C9—Cl3	118.18 (14)
H8—N2—H11	105 (3)	C9—C10—C11	121.09 (19)
C2—C1—C6	122.87 (18)	C9—C10—H2	117.1 (15)
C2—C1—Cl1	119.93 (16)	C11—C10—H2	121.8 (15)
C6—C1—Cl1	117.20 (15)	C10—C11—C12	118.14 (18)
C1—C2—C3	120.23 (19)	C10—C11—C15	120.22 (18)
C1—C2—H5	123.4 (15)	C12—C11—C15	121.64 (17)
C3—C2—H5	116.1 (15)	C13—C12—C11	120.04 (17)
C4—C3—C2	118.29 (18)	C13—C12—H1	120.7 (11)
C4—C3—C7	119.86 (19)	C11—C12—H1	119.2 (11)
C2—C3—C7	121.82 (18)	C12—C13—C14	123.08 (17)
C5—C4—C3	120.33 (19)	C12—C13—Cl4	118.96 (15)
C5—C4—H3	121.3 (15)	C14—C13—Cl4	117.93 (15)
C3—C4—H3	118.4 (15)	C9—C14—C13	115.17 (16)
C4—C5—C6	123.30 (18)	C9—C14—N2	121.63 (17)
C4—C5—Cl2	119.27 (15)	C13—C14—N2	123.02 (18)
C6—C5—Cl2	117.44 (15)	O2—C15—C16	107.45 (18)
N1—C6—C5	122.21 (18)	O2—C15—C11	112.01 (18)
N1—C6—C1	122.73 (18)	C16—C15—C11	111.70 (19)
C5—C6—C1	114.97 (17)	O2—C15—H4	109.2 (14)
O1—C7—C8	106.7 (2)	C16—C15—H4	106.3 (13)
O1—C7—C3	111.71 (19)	C11—C15—H4	109.9 (14)
C8—C7—C3	111.63 (19)	C15—C16—H6	109.3 (17)
O1—C7—H12	108.6 (13)	C15—C16—H9	108.2 (13)
C8—C7—H12	107.1 (13)	H6—C16—H9	112 (2)
C3—C7—H12	110.9 (13)	C15—C16—H10	106.3 (16)
C7—C8—H8A	109.5	H6—C16—H10	113 (2)
C7—C8—H8B	109.5	H9—C16—H10	108 (2)

### Hydrogen-bond geometry (Å, °)

**Table:**

*D*—H···*A*	*D*—H	H···*A*	*D*···*A*	*D*—H···*A*
N1—H13···O2^i^	0.82 (3)	2.17 (3)	2.927 (3)	154 (3)
O1—H16···N1^ii^	0.83 (3)	2.12 (3)	2.941 (3)	169 (3)
O2—H18···N2^iii^	0.79 (3)	2.13 (3)	2.912 (3)	172 (2)

Symmetry codes: (i) *x*+1, −*y*, *z*+1/2; (ii) −*x*+2, −*y*, −*z*+2; (iii) −*x*+1, −*y*, −*z*+1.
